# 肺癌患者恶病质的药物治疗与营养支持研究进展

**DOI:** 10.3779/j.issn.1009-3419.2022.101.21

**Published:** 2022-06-20

**Authors:** 杰敏 王, 维慧 贾, 丹阳 厉, 艳梅 宋, 宁鑫 孙, 科 杨, 洪利 李, 崇高 尹

**Affiliations:** 1 261053 潍坊，潍坊医学院护理学院 Colloge of Nursing, Weifang Medical University, Weifang 261053, China; 2 261053 潍坊，潍坊医学院医学研究实验中心 Medical Research Center, Weifang Medical University, Weifang 261053, China

**Keywords:** 肺肿瘤, 恶病质, 药物治疗, 营养支持, 多模式治疗, Lung neoplasms, Cachexia, Drug therapy, Nutritional support, Combined modality therapy

## Abstract

恶病质是肺癌患者的一种常见并发症，它会加重化疗毒副作用、阻碍治疗计划、削弱化疗反应性、降低生活质量，增加并发症及死亡率，严重危害肺癌患者的生理、心理健康。肿瘤恶病质的发病原因与发病机制都极为复杂，使其治疗具有艰巨性与复杂性。控制肺癌患者的恶病质需要采取抗肿瘤治疗、抑制炎症反应、营养支持、体育锻炼、缓解症状等多种手段，发挥多模式治疗的协同作用对抗肿瘤恶病质的多种机制。迄今为止，学科内已就任何单一疗法都不能控制恶病质的发生发展达成了一致共识。有些疗法取得了一定的研究进展，但需要在充分评估肺癌患者个体特征后结合多模式治疗实施。本文重点综述了肿瘤恶病质的药物治疗与营养支持等干预方法在肺癌患者人群中的应用，并对肺癌患者恶病质控制的研究方向进行展望。

## Abstract (en)

Cachexia is a common complication in patients with lung cancer. It aggravates the toxic and side effects of chemotherapy, hinders the treatment plan, weakens the responsiveness of chemotherapy, reduces the quality of life, increases complications and mortality, and seriously endangers the physical and mental health of patients with lung cancer. The causes and pathogenesis of tumor cachexia are extremely complex, which makes its treatment difficult and complex. Controlling cachexia in lung cancer patients requires many means such as anti-tumor therapy, inhibition of inflammatory response, nutritional support, physical exercise, and relief of symptoms to exert the synergistic effect of multimodal therapy against multiple mechanisms of tumor cachexia. To date, there has been a consensus within the discipline that no single therapy can control the development of cachexia. Some therapies have made some progress, but they need to be implemented in combination with multimodal therapy after fully assessing the individual characteristics of lung cancer patients. This article reviews the application of drug therapy and nutritional support in lung cancer patients, and looks forward to the research direction of cachexia control in lung cancer patients.

肿瘤恶病质是一种以持续性的骨骼肌肉丢失，伴或不伴脂肪组织丢失为特征的代谢综合征^[1]^。肿瘤恶病质的病理生理学机制复杂，由炎症反应、营养物质代谢紊乱、神经激素变化以及治疗过程中的不良反应等多种因素作用导致。肿瘤恶病质在肺癌患者中的发生率为36%-76%^[2]^，多见于晚期肺癌患者，22%的肺癌患者最终因恶病质而死亡^[3]^。恶病质会降低肺癌患者的生存质量，限制治疗计划，缩短肺癌患者的总生存期，是造成肺癌患者不良预后的因素之一^[4, 5]^。通过对专门针对肺癌患者或纳入了肺癌患者的研究进行总结，目前用于改善恶病质的干预措施包括药物、营养支持、体育锻炼、心理社会支持或以上干预措施的各种组合，采用体重、肌肉量、营养状况、身体功能、生活质量等指标对效果进行评价。而就目前的研究结果来看，任何一种单一的疗法都不能有效地阻止恶病质的进展，本文结合国内外肿瘤恶病质治疗指南，重点对以药物治疗或营养支持为主的肺癌恶病质干预方法进行综述，并基于此对现阶段被广泛认同的多模式治疗进行概述。

## 1 药物治疗

### 1.1 孕激素制剂

研究^[6, 7]^表明，以甲地孕酮（megestrol acetate, MA）和甲羟孕酮（medroxyprogesterone acetate, MPA）为代表的孕激素类药可通过抑制肿瘤坏死因子的释放以增进食欲，还能促进机体蛋白质及脂肪合成，增加患者的体重，从而改善恶病质。MA是最早用于研究治疗恶病质的药物之一，美国食品药品监督管理局（State Food and Drug Administration, SFDA）于1993年便将其批准作为治疗恶病质的临床标准用药，美国肿瘤护理学会（Oncology Nursing Society）、美国国家综合癌症网络（National Comprehensive Cancer Network, NCCN）指南也都对MA和甲羟孕酮用于治疗恶病质作出了明确推荐^[8, 9]^。值得注意的是，MA未被证实可以增加瘦体重，所引起的体重增加主要来自于体脂含量和体细胞体积增加。MA是一种相对无毒性的药物，安全性较好，不良反应发生率低，但应用时仍要注意潜在的体液潴留、血栓栓塞等副作用，可以同时服用抗凝血药物如小剂量阿司匹林等。此外，沙利度胺能够有效抑制肿瘤坏死因子（tumor necrosis factor, TNF）-*α*的释放，改善恶病质患者的厌食症状，增加去脂体重^[10, 11]^。在治疗肺癌患者恶病质时，沙利度胺与MA联合使用比单独使用MA更有效^[12]^。但是，沙利度胺具有多种不良反应，且在不同的个体中治疗效果不稳定，因此难以论定沙利度胺对于改善肺癌患者的恶病质是否安全有效。

### 1.2 糖皮质激素

美国临床肿瘤学会（American Society of Clinical Oncology, ASCO）在指南中将糖皮质激素作为恶病质患者刺激食欲推荐用药^[13]^。地塞米松具有长效和廉价的特点，是肿瘤治疗常用的糖皮质激素类药物。研究表明，地塞米松的使用可使晚期癌症患者的恶病质、疲劳、厌食、抑郁症状显著改善^[14]^，与5-HT3受体抑制剂联用还可缓解化疗导致的恶心呕吐^[15]^。在接受化疗的肺癌患者中，地塞米松对体重和食欲有保护作用^[16]^。然而，糖皮质激素仅可短期提供给晚期癌症患者，长期应用糖皮质激素会出现诸多不良反应，如高血糖、感染、免疫力下降等，甚至还会影响体内蛋白质代谢合成引起肌肉萎缩，从而加重恶病质的进展。因此，欧洲姑息治疗研究合作组制定的恶病质指南中建议，恶液质患者使用糖皮质激素以改善食欲及生活质量时，推荐疗程为不超过2周^[17]^。此外，晚期肺癌患者可能存在糖皮质激素的禁忌证，使用前应根据治疗目标充分权衡风险与获益。

### 1.3 生长激素释放肽

有研究表明，生长激素释放肽可能在肺癌患者恶病质的发病机制中起作用^[18]^。生长激素释放肽由胃黏液腺分泌，其生理功能为刺激生长激素的释放，通过刺激食欲、影响脂肪酸代谢等机制实现改善恶病质的作用^[19, 20]^。阿拉莫林是一种新型生长激素释放肽受体激动剂，作用机制与生长激素释放肽相同，具有可以口服、半衰期长的优点。阿拉莫林已多次在肺癌恶病质患者中进行了临床试验，证明阿拉莫林可以改善患者的瘦体重、握力、体重和生活质量以及长期使用的安全性^[21, 22]^，最常见的不良反应是高血糖，发生率约1%^[23]^。

### 1.4 其他

大麻素、雄激素以及部分非甾体抗炎药等药物也有潜力能够改善肺癌患者的恶病质。大麻素的活性成分为屈大麻酚，具有止吐、刺激食欲等作用。有研究^[24]^将大麻酚合成物Nabilone应用于肺癌患者中，发现Nabilone可以增加肺癌患者的热量摄入，改善患者的生活质量，并且安全性较高。但要注意大麻素在使用时可能的副作用如精神不良反应。有研究^[25]^发现选择性雄激素受体调节剂在改善化疗非小细胞肺癌患者的瘦体重和肌肉功能方面有良好疗效。炎症反应是恶病质的重要病理生理学驱动因素，因此，减轻炎症反应被认为是恶病质治疗的关键部分。非甾体抗炎药价格低廉、易于管理，塞来昔布是在肿瘤恶病质中研究最多的抗炎药物之一，已被证明有利于保持体重、运动状态和肌肉力量以及相对较少的副作用^[26]^。目前还缺乏充足的证据就以上药物的疗效与安全性得出可靠结论，需要进行更大规模的临床试验。

## 2 营养支持

### 2.1 肠内/肠外营养

欧洲肠外肠内营养学会（European Society for Parenteraland Enteral Nutrition, ESPEN）制定的肿瘤学临床营养指南支持使用营养干预预防和治疗恶病质^[26]^，但对于晚期肺癌恶病质患者来说，关键问题在于实施营养干预的最佳时机，不同恶病质分期的患者对营养支持的反应和获益有差异^[27]^，需要充分考虑患者耐受情况后再进行实施。晚期肺癌患者恶病质的机制与饮食和营养不足导致消瘦的机制完全不同，这种由肿瘤细胞带来的静息能量高耗和能量剥夺无法靠单纯的营养支持抵消。此外，肠内营养的并发症较多，严重时会引起代谢紊乱、感染、再喂养综合征等。对于严重吸收不良或体能衰弱的患者来说，在给予肠内营养之前应先进行一段时间的肠外营养治疗，以改善其肠道酶的活性及黏膜细胞状态^[28]^。当恶病质患者无法接受肠内营养时，可以使用全肠外营养或部分肠外营养进行营养支持。对于晚期肺癌患者尤其是难治期恶病质患者，需要慎重使用肠外营养。在晚期癌症恶病质患者中，肠外营养既不能改善生活质量，也不能提高生存率，相关并发症可能会高于其应用价值^[29]^。

### 2.2 营养指导

营养指导用于改善肺癌患者的能量和蛋白质摄入已被证实具有良好的效果和依从性。营养指导通常由注册营养师对肺癌患者的病情与病史、饮食偏好、营养状况和影响饮食摄入的相关因素（食欲、咀嚼能力、吞咽能力、味觉障碍、社会环境因素等）进行评估，并以口头、书面等形式向患者提供如何提高能量和蛋白质摄入的建议，有时也会向患者的照顾者提供咨询^[30-32]^。一般来说，指导由专业营养师给予，建议增加进餐频率和进食能量密集的食物，添加富含能量和蛋白质的营养补充剂，每日摄入 > 1.0 g/kg的蛋白质^[26]^。这些营养指导使患者在治疗期间相比没有接受营养指导的对照组而言保持了更稳定的体重，改善了营养状况，保持了稳定的生活质量。肺癌患者能够从营养指导中获益，心理与社会支持也可以帮助患者形成饮食行为上的良好转变^[33]^。

### 2.3 口服营养补充剂（oral nutritional supplements, ONS）

有研究^[34]^在患者因为恶心呕吐等治疗不良反应导致饮食摄入减少时，会使用口服营养补充剂作为平常膳食的补充。常用的口服营养补充剂有鱼油或二十碳五烯酸（eicosapentaenoic acid, EPA）、支链氨基酸（branched-chain amino acids, BCAA）、乳清蛋白等^[32, 35, 36]^。鱼油是一种富含EPA和二十二碳六烯酸（docosahexaenoic acid, DHA）等ω-3脂肪酸的油脂。有研究发现EPA+DHA用于肺癌患者具有抗炎、抗氧化作用，可以改善患者的营养状况^[37]^。有些患者难以接受液体ONS的口感，提供EPA/DHA液体或鱼油胶囊可以保证更好的依从性。总的来说，由于癌症患者存在个体差异性，一对一制定营养策略的效果要优于使用ONS作为标准化饮食干预的效果^[38]^。EPA单独用于改善恶病质状况的证据尚不充分，多与其他药物或干预方法联合用于治疗肿瘤恶病质。一项III期临床试验^[39]^证明联用甲羟孕酮或甲地孕酮、EPA、左旋肉碱和沙利度胺有良好的改善肿瘤恶病质的效果，且具备较高的安全性。EPA与甲地孕酮联合治疗癌症患者恶病质也在国内《肿瘤恶液质临床诊断与治疗指南》中作为共识性建议予以推荐^[40]^。BCAA是人体必需氨基酸亮氨酸、异亮氨酸和缬氨酸的统称，是蛋白质合成和降解的调节因子，富含亮氨酸的补充剂比普通食物更有效地促进肌肉蛋白质合成^[41]^。可以选择高亮氨酸配方的乳清蛋白补充剂，其吸收利用率高，产生的肠胃负担轻，可以保证良好的依从性和安全性，与体育锻炼干预结合能够显著改善晚期癌症患者的握力^[36]^。

## 3 多模式治疗

对于肺癌恶病质的治疗现已达成的共识是：单独使用药物、营养支持或体育锻炼都不足以稳定或逆转这种复杂机制引起的多因素综合征^[42]^。三者结合的多模式治疗可能通过同时作用于多种机制使肺癌患者获益并且能为肺癌患者提供全面的生活方式调整^[43]^。许多研究^[30, 32, 34, 35, 44, 45]^针对肿瘤恶病质探寻了多种治疗方案，通常包含有药物、营养指导、富含蛋白质或EPA的口服营养补充剂以及有氧结合抗阻训练的体育锻炼计划。在肺癌患者人群中进行的试验证明了多模式治疗的可行性与安全性^[30]^。对于肺癌患者恶病质的干预效果方面，多模式治疗可以增加饮食摄入、减轻恶心呕吐、增加体重和骨骼肌质量、提高机体功能状态、提高生活质量^[32, 34, 36, 44, 45]^。晚期肺癌患者病情变化快、症状负担大、表现状态差，因此多模式治疗并不适用于所有患者。鉴于人群在诊断和治疗上的异质性，同样的干预方案也不能达到相同程度的效果。恶病质早期的患者更容易从恶病质治疗中获益^[46]^，这提示我们应在肿瘤治疗的早期就采取积极的措施。此外，社会心理支持是癌症恶病质患者仍然未得到满足的需求，应当被纳入多模式治疗框架^[47]^。

## 4 小结

肺癌患者的恶病质目前仍没有明确的解决方法，而恶病质的发生和发展会严重威胁患者的治疗与生存，因此这种缺乏治疗突破的困境亟待解决。孕激素、糖皮质激素、非甾体类抗炎药对肺癌患者的恶病质有一定的治疗作用。一些有前景的药物如阿拉莫林也正在开发中。但这些药物应该在全面的多模式治疗的基础上提供。

综上所述，治疗肺癌患者恶病质的最佳策略应是加强治疗维度的全面性，包括控制炎症反应、增加营养摄入、锻炼肌肉力量、加强心理社会支持等多种干预的组合。全方位地对患者进行个体化评估也十分重要。本文参考的研究仍具有一定的局限性，主要是由于肺癌患者的疾病因素和治疗因素所致的样本量小、样本异质性大、随访时间短、失访率高、依从性不佳等。针对肺癌患者恶病质的治疗还需要更大规模的临床试验来验证疗法的效果和安全性，也需要通过对患者人群的不断细分进一步研究不同原因、不同特征的恶病质患者的治疗策略。
